# A Systematic Review of Essential Oils and the Endocannabinoid System: A Connection Worthy of Further Exploration

**DOI:** 10.1155/2020/8035301

**Published:** 2020-05-15

**Authors:** Scott A. Johnson, Damian Rodriguez, Kathryn Allred

**Affiliations:** dōTERRA International, LLC, 389 S. 1300 W, Pleasant Grove, UT 84062, USA

## Abstract

Aromatic compounds have a long history of use as medicines in most recorded cultures. An increasing interest in these therapeutic volatile molecules in both scientific and lay communities has led to the advancement of essential oils as phytomedicines. Recent discoveries suggest essential oils augment the endocannabinoid system in a positive manner to mitigate various pathologies. However, the exact mechanisms whereby essential oils influence endocannabinoid system activity are not fully known, these studies provide a glimpse into their involvement and warrant further evaluation. Additional study of the interaction between essential oils and the endocannabinoid system may lead to promising phytomedicines for the treatment of diseases and conditions involving dysregulation or activation of the endocannabinoid system.

## 1. Introduction

Aromatherapy is one of the most widely used integrative therapies in the world, with a rich tradition dating back centuries. Volatile aromatic compounds distilled or expressed from plants—leaves, flowers, seeds, bark, resins, and more—called essential oils are used medicinally for a variety of ailments. Volatile extracts produced by solvent extraction are not considered true essential oils. One exception may be volatile concentrates extracted with carbon dioxide (typically in a supercritical state), which can produce essential oils very similar to traditionally distilled or expressed essential oils without adding any solvent to the end product. Supercritical carbon dioxide extraction of aromatic molecules can be either “select” or “total.” Select extracts frequently mimic traditionally distilled essential oils in chemical composition, whereas total extracts contain large amounts of nonvolatile constituents in them and are dissimilar to their traditionally obtained counterparts. These extracts are highly purified and concentrated with aromas authentic to the original plant materials. Despite the similarity between supercritical select extracts and traditionally distilled essential oils, they are not universally accepted as essential oils and many prefer to distinguish them as plant extracts rather than essential oils.

Essential oils are complex mixtures of volatile aromatic compounds mainly composed of terpenes biosynthesized by the mevalonate pathway [1]. Furthermore, they contain phenolic compounds derived from the shikimate pathway. These therapeutic compounds can be absorbed through the olfactory system (inhalation), through the skin (topically), or by oral administration [2]. Less commonly, they are administered transmucosally via the rectum or vagina. Plant essential oils contain lipophilic volatiles that freely cross cellular membranes. Once absorbed, their chemical diversity, low molecular weight, and structure allow them to produce a wide spectrum of activity through multiple pathways, mechanisms, and pharmacological targets.

A large body of evidence describes the effects of odors on the human brain and emotions. Essential oil molecules are uniquely qualified to influence mood, alertness, stress, anxiety, and task performance because of their direct connection to areas of the brain involved in emotions and cognition, especially the limbic system [3]. Studies evaluating the effects of essential oils on human physiology and specific disease states are also growing, but much of this to date has been performed in vitro or animal research. Hundreds of clinical trials have also investigated essential oils, with the majority occurring in the last decade. Nevertheless, multiple promising properties have been confirmed—anxiolytic, antidepressant, anti-inflammatory, analgesic, hormonogenic, antidiabetic, antitumoral, immunomodulatory, and more—that await confirmation in randomized, controlled, and blinded human trials [3].

A growing interest in the endocannabinoid system (ECS) and its effects on human health and physiology has emerged as a consequence of relaxing legislative restriction on *Cannabis* spp. plant extracts and their active constituents, such as cannabidiol (CBD) and Δ9-tetrahydrocannabinol (THC). The ECS is a system of enzymes (that control the production and degradation of endogenous cannabinoids), cannabinoid receptors (CB1R and CB2R), and molecules that interact with these receptors. Cannabinoid receptors type 1 (CB1R) are predominantly expressed in the central nervous system (CNS), making them a potential target for neuropsychological disorders and neurodegenerative diseases [4–6]. The primary drawback to molecules that bind to CB1R is their psychoactive potential, which may limit therapeutic use [7]. Indeed, activation of CB1R may, in some cases, precipitate episodes of psychosis and panic [7]. On the other hand, inhibiting CB1R could produce depressive or anxiety-related behaviors. All of this needs to be taken into account when treating individuals through exploitation of the endocannabinoid system.

On the contrary, cannabinoid receptor type 2 (CB2R) are widely expressed in the immune system and periphery. CB2R is a promising target for inflammatory conditions, neuropathic pain, and immune modulation [8, 9]. Activation or upregulation of CB2R induces receptor conformational rearrangements that can affect myriad signaling pathways, which is vital for therapeutic activity [8]. They have been the subject of considerable attention primarily due to their promising therapeutic potential while avoiding undesirable psychotropic effects associated with CB1R agonists.

Cannabinoids can modulate immune cell function, activity, and secretion of cytokines [10, 11]. Compounds that activate CB2R are of particular interest for their immunomodulatory activity because these receptors are predominantly expressed in the immune system. CB2R is expressed in human leukocytes, B cells, basophils, dendritic cells, eosinophils, mast cells, macrophages, microglia, monocytes, natural killer cells, neutrophils, platelets, and T cells, highlighting its potential in immune modulation [9]. The complexity of the immune system means it has ample opportunity to fall out of balance and be under- or overactive. The role of the ECS in immune regulation—overall function, tone, and activity—is very important. Inflammation plays a crucial role in the immune system's defensive response to harmful stimuli, such as pathogens and injury. Autoimmune diseases frequently involve a chronic hyperinflammatory response. Endogenous cannabinoids can modulate inflammation both through direct cannabinoid receptor binding and other mechanisms [12]. Emerging evidence shows that phytocannabinoids also exert regulatory control over immune and inflammatory responses [13–16]. It is likely that the subtle but significant role of the ECS in immune regulation can be exploited in the management of human disease.

Both CB1R and CB2R are distributed in various organs and tissues throughout the body, affecting every major organ of the body and whose expression changes in disease states [17]. It is well known that these receptors and the molecules that interact with them have a significant signaling and regulatory effect on cells, tissues, organs, and organ systems [18]. Indeed, the ECS is vital for human survival and plays a critical role in maintaining homeostasis so much so that some consider it the “master regulator” of the human body [19].

Given the interest in both EOs and the ECS, a systematic review was conducted to determine current evidence for use of EOs as therapeutic agents for conditions involving dysregulation of the ECS.

## 2. Methods

### 2.1. Systematic Review

The present review employed a systematic search of the National Institute of Health PubMed database for all articles containing the keywords “essential oil,” “aromatherapy,” and “beta-caryophyllene” together with either of three key words or phrases “endocannabinoid system,” “endocannabinoid,” or “cannabinoid.” Only primary research articles published in the English language between 1960 and 2020 (up to January 10, 2020) with relevant information on EOs that modulate endocannabinoid system activity were included in the review. Publications documenting the activity of methanolic extracts or aqueous extracts were excluded.

Of the 20 research articles identified in the initial search, three were excluded as they primarily mentioned beta-caryophyllene as a cannabinoid and are not relevant to EO modulation of the ECS. A total of 17 articles meeting the inclusion criteria were systematically reviewed and the research documented for ECS modulation.

Research directly related to the subject matter was in vitro and animal in vivo. No clinical trials have been conducted on EOs and ECS modulation.

## 3. Results

### 3.1. Beta-Caryophyllene

Widely distributed in essential oils, black pepper (*Piper nigrum*), melissa (*Melissa officinalis*), guava leaf (*Psidium guajava*), hemp (*Cannabis sativa*), clove (*Eugenia caryophyllata*), and ylang ylang (*Cananga odorata*), but most abundantly found in copaiba (*Copaifera* spp.; up to 87.3%) [20], beta-caryophyllene (BCP) is a sesquiterpene alkene and atypical dietary cannabinoid [21–25]. In nature, BCP is frequently found together with small quantities of its isomers, isocaryophyllene, and alpha-humulene or in mixture with its oxidation product caryophyllene oxide. BCP is well researched and shares similar properties (e.g., analgesic and anti-inflammatory) to other cannabinoids, which allow it to initiate similar physiological responses in the ECS. It is a full selective agonist of CB2R, allowing for a therapeutic action without the psychoactive effects of cannabinoids that bind to CB1R [26, 27]. BCP is considered nontoxic with a wide safety margin [28]. It is considered a functional nonpsychoactive, polypharmacological dietary cannabinoid and anti-inflammatory agent with a demonstrated activity on a number of receptor targets in the human body [29]. Essential oils that contain BCP have a direct action on the ECS, as BCP binds to CB2R.

**Figure 1:**
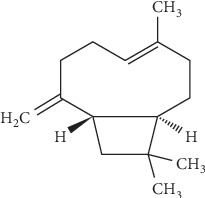
Chemical structure of beta-caryophyllene.

BCP (see its chemical structure in Figure 1) is an attractive compound for chronic pain (both inflammatory and neurogenic) due to its direct action on CB2R without creating a tolerance, as demonstrated in an animal model of neuropathic pain [30]. Remarkably, the researchers concluded that the effect of BCP became stronger during the treatment period. This is significant considering drug tolerance after prolonged use is observed with other analgesic therapies and may require increased dosage, which may accelerate tolerance and further reduce the effects of a therapeutic substance. BCP exerts potent anti-inflammatory effects when taken orally, which may be explained by its ability to simultaneously inhibit pathways (toll-like receptor complex CD14/TLR4/MD2) that increase the production of proinflammatory cytokines (IL-1*β*, IL-6, IL-8, and TNF-*⍺*), reduce immune-related inflammation, and synergize the *µ*-opioid receptor pathways [28, 31]. Preclinical models demonstrate that BCP can reduce inflammation in a variety of bacterial-caused, eosinophil-related, and neuropathic inflammatory conditions [32]. It can also enhance the effects of other pain medications like morphine, making it a promising candidate to reduce effective drug dose and therefore potential drug dependence and tolerance [33]. In this way, it modulates pain signaling pathways in a manner that may enhance, and not compete with, the benefits of other pain-or inflammation-relieving substances.

**Figure 2:**
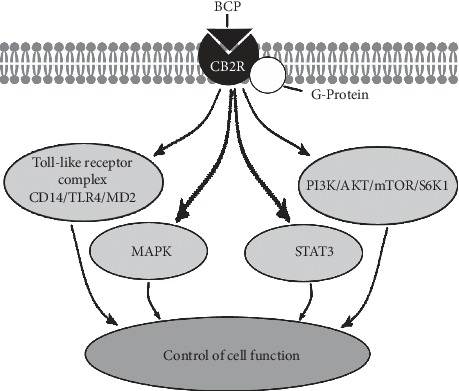
Beta-caryophyllene exerts its effects partly by binding to CB2R in the ECS. The G-protein-coupled CB2R signals multiple different cellular pathways to modulate cellular function and activity. For clarity, crosstalk between signaling pathways has been omitted.

BCP modulates multiple pathways involved in cancer pathogenesis including MAPK, PI3K/AKT/mTOR/S6K1, and STAT3 (Figure 2), activates cytotoxic pathways against tumors, potentiates pathways that hinder metastasis, reduces the expression of oncogenes and proteins, and simultaneously increases genes and proteins that destroy cancer cells [34–37]. Laboratory studies suggest BCP has promise against kidney, lung, colorectal, liver, oral, melanoma, leukemia, lymphoma, and neuroblastoma cancers and possesses chemopreventive properties [32, 38, 39].

BCP shows promise for treating depression and stress related mental illnesses due to its direct binding to CB2R, which modulates ECS activity (Table 1) [40]. The ECS controls cognitive and emotional responses to stressors through CBR interactions. In addition, preclinical research suggests BCP is a neuroprotective, antidiabetic, antioxidant, and anticonvulsive agent, reduces neuroinflammation, improves lipid profiles, alleviates endometriosis, exhibits promise for interstitial cystitis, is helpful for substance abuse disorders, and protects against nonalcoholic fatty liver disease [13, 32, 41–53].

### 3.2. Lavender (*Lavandula angustifolia*) Essential Oil

Lavender is one of the most popular and widely studied essential oils. It is primarily composed of linalool (25%–45%) and linalyl acetate (25%–46%) but also contains moderate levels of beta-caryophyllene, (Z)-beta-ocimene, (E)-beta-ocimene, and lavandulyl acetate [54–56]. It is frequently used in aromatherapy for the skin, anxiety, stress, pain, and to improve sleep [57].

Neuropathic pain is one of the most difficult conditions to treat, and more effective treatment options are urgently needed. A 2019 study investigated the benefits of oral administration of lavender (36.0% linalyl acetate, 35.3% linalool, 3.4% beta-caryophyllene) for neuropathic pain in mice [58]. Mice with spared nerve injury were administered 100 mg/kg body weight as an acute dose, which alleviated SNI-induced mechanical allodynia with efficacy comparable to the reference drug pregabalin.

**Figure 3:**
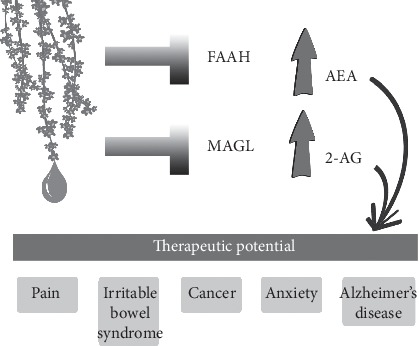
As a dual blocker of FAAH and MAGL, lavender essential oil is a promising treatment for a variety of conditions, including pain, irritable bowel syndrome, cancer, anxiety, and Alzheimer's disease.

Further investigation of the mechanism of action by the study authors found that lavender essential oil markedly decreased the phosphorylation of ERK1, ERK2, and JNK1 and decreased the levels of iNOS in the spinal cord. Additionally, lavender essential oil inhibited the endocannabinoid degradation enzymes FAAH (fatty acid amide hydrolase) and MAGL (monoacylglycerol lipase), particularly FAAH (Table 1). FAAH and MAGL can synthesize or degrade endocannabinoids depending on body needs. FAAH inhibition is beneficial in CNS and pain disorders due to conservation of endocannabinoids and enhancement of endocannabinoid signaling [59, 60]. Elevation of endocannabinoids causes marked antinociception in neuropathic pain models [61, 62]. The signaling function of the endogenous cannabinoid N-arachidonoylethanolamide (AEA; also known as anandamide) is terminated by enzyme hydrolysis principally involving FAAH, which directly affects specific brain regions related to emotionality, behavior planning, and motivation (hippocampus, striatum, and frontal cortex) [63, 64]. The blockade of FAAH and MAGL enzymatic activity is considered a therapeutic target for neuropathic pain relief and may also alleviate inflammatory bowel diseases, anxiety, cancer metastasis, and Alzheimer's diseases (Figure 3) [65, 66]. A primary advantage of FAAH inhibitors is their modulation of the ECS without psychotropic effects. This research highlights the need to investigate other essential oils with demonstrated analgesic and antinociceptive activity and whether this activity is related to endocannabinoid enzyme blockade.

### 3.3. Atlas Cedarwood (*Cedrus atlantica*) Essential Oil

Atlas cedarwood is typically very rich in sesquiterpenes (primarily himachalenes) and frequently used in aromatherapy for nervous disorders like anxiety and stress. Research shows that Atlas cedarwood possesses analgesic, anti-inflammatory,anti-leukemic, and insecticidal properties [67–70].

Italian researchers who previously discovered the analgesic activity of cedarwood via inhalation in a model of postoperative pain designed an additional study to identify its mechanism of action [71]. Male Swiss mice were subject to plantar incision. CB1R and CB2R antagonists (AM281 and AM630) were administered in different sites (intraperitoneal, intraplantar, and intrathecal), and the FAAH and MAGL endocannabinoid degradation inhibitors (URB937 and JZL184) were employed to determine ECS system involvement.

Cedarwood essential oil (46.6% beta-himachalene, 16.6% alpha-himachalene, and 10.4% gamma-himachalene) was inhaled by positioning 200 *μ*L of the oil at the bottom of the cage in a Petri dish. Incised mice were then introduced into the cage for 30 minutes of inhalation. CBR antagonists were administered twenty minutes prior to exposure to the oil. Animals were also pretreated with FAAH and MAGL inhibitors 1.5 hours prior to cedarwood inhalation to test if inhibition of these enzymes was involved in cedarwood's antihyperalgesic effects.

**Figure 4:**
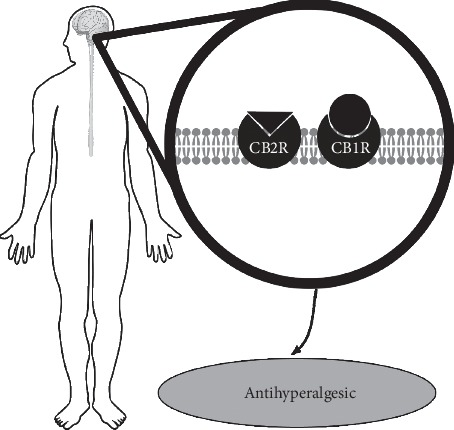
The antihyperalgesic activity of cedarwood essential oil involves activation of both CB1R and CB2R within the central nervous system.

Inhalation of cedarwood produced a significant antihyperalgesic effect. Pretreatment with CB1R and CB2R antagonists prevented the antihyperalgesic activity of cedarwood oil inhalation. The observed effects show that the antihyperalgesic effects of cedarwood oil inhalation are mediated through the activation of central CB1R and CB2R (Table 1 and Figure 4) but not peripheral receptors. Inhibition of FAAH and MAGL prolonged the antihyperalgesic effects of cedarwood inhalation with activity occurring as early as 30 minutes and remaining for up to six hours after exposure. This study demonstrates that cedarwood essential oil reduces postoperative hyperalgesia through activation of descending pain modulation pathways via activation of CNS pathways (opioidergic, serotonergic, noradrenergic, and dopaminergic) and activation of central CBR.

The findings are interesting since no known phytocannabinoid or CBR ligand is present in cedarwood essential oil. BCP may be present in trace amounts occasionally, but it is not always present and was not reported in this study as a constituent of the oil. This suggests that *Cedrus atlantica* essential oil may stimulate the release of endocannabinoids or inhibit their degradation by enzymes to enhance regulation of homeostasis by the ECS, and further research is warranted to investigate if other essential oils have similar effects.

### 3.4. Mastic (*Pistacia lentiscus*) Essential Oil

Mastic leaf essential oil is obtained via supercritical carbon dioxide extraction or traditional steam distillation from the gum resin, leaves, or fruits. Perhaps due to its low yield, it is less commonly used in aromatherapy. However, mastic oil is used in aromatherapy and both mastic extract and the essential oil are used for their antihypertensive, anti-inflammatory, analgesic, antiseptic, and respiratory supportive properties, as well as in the treatment of gastric disorders [72–74]. Its composition varies widely according to the published research [2].

Mastic leaf essential oil (a carbon dioxide select extract) consisting of 19.9% germacrene D, 8.7% gamma-cadinene, 6.6% beta-caryophyllene, 6.3% alpha-pinene, 5.8% sabinene, 4.2% delta-cadinene, 3.9% myrcene, and 3.7% beta-phellandrene was evaluated for protection against brain tissue damage caused by an ischemia/reperfusion injury [75].

Cerebral ischemia is caused by occlusion of internal carotid artery, which interrupts brain circulation and prevents cerebral tissue from maintaining basal metabolism. Following this occlusion, a cascade of interconnected pathophysiological events occurs that alters the structure and functional organization of the brain [76, 77]. Reperfusion of neural ischemic tissue after occlusion leads to oxidative stress that can eventually cause neuronal death [78]. Furthermore, altered endocannabinoid levels have been observed in people with acute ischemic stroke, likely as an adaptive mechanism due to neuronal death [79, 80]. Moreover, increased expression of CB2R is noted in the brainstem, microglia, and astrocytes after CNS insults, such as neuroinflammation [81–83]. Given its potent anti-inflammatory activities, CB2R activation has been proven to reduce postischemic inflammation [84]. In addition, CB2R have the potential to restrain the inflammatory process that contributes to the decline of neural function in neurodegenerative disease [85].

**Figure 5:**
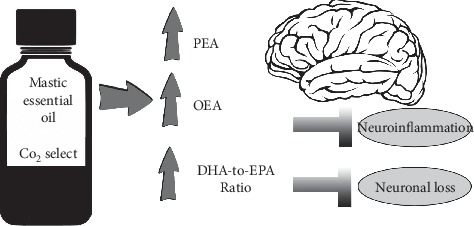
Mastic increases cannabinoid congeners PEA and OEA and improves DHA-to-EPA ratio, which preserves brain structure and reduces neuroinflammation and neuronal loss caused by stroke.

In the aforementioned study evaluating mastic essential oil against brain tissue damage, male Wistar rats were subject to transient bilateral common carotid artery occlusion followed by reperfusion. Gavage administration of 200 mg of mastic leaf essential oil (containing about 60 mg/kg bodyweight of BCP) in 0.45 mL sunflower oil occurred six hours prior to ischemia. Analysis of the frontal cortex and the remaining posterior cortex of rats showed that brain DHA levels were preserved in rats pretreated with mastic essential oil. An increase in the DHA-to-EPA ratio and significantly increased levels of cannabinoid congeners palmitoylethanolamide (PEA) and oleoylethanolamide (OEA) were also observed (Table 1 and Figure 5). An increased DHA-to-EPA ratio may indicate an increased metabolism of omega-3 fatty acids to supply damaged brain tissue with newly produced DHA or prompt metabolism of EPA to DHA due to augmented EPA entry into the brain. Cyclooxygenase-2 (COX-2) levels were significantly decreased in the frontal cortex in rats pretreated with mastic essential oil compared with control, suggesting less neuroinflammation. No change was observed in levels of two endocannabinoids, AEA, or 2-arachidonoyl-monoacylglycerol (2-AG).

The present study demonstrates that a single dose of mastic essential oil is sufficient to mitigate stroke damage by modifying ECS congener profiles. Altogether, the findings suggest a preservation of brain structure and reduced neuroinflammation and neuronal loss.

A possible explanation of the observed ECS effects is the presence of moderate amounts of BCP. BCP prevents activation of the ECS after ischemia reperfusion injury, spares DHA levels, and prevents increases in COX-2 and peroxisome-proliferator activated receptor-alpha (PPAR-alpha) protein levels [86]. The same research showed that BCP significantly reduces AEA levels (−56%; post hoc adjusted *p* < 0.0001) after ischemia reperfusion injury. The evidence suggests BCP induces positive modulation of the ECS and lipid peroxidation to control damage caused by hypoperfusion/reperfusion-induced oxidative stress injury. Nevertheless, synergistic effects by other aromatic volatile compounds, some of which have demonstrated antioxidant and anti-inflammatory activity, cannot be ruled out.

### 3.5. Chir Pine (*Pinus roxburghii*) Essential Oil

Chir pine is an evergreen, coniferous tree native to the Himalayas. Essential oil can be extracted from various parts of the tree, including the needles, bark, wood, and cones. Depending on the plant parts used and other factors, chir pine essential oil produces essential oil with a variety of compositions and major aromatic compounds. It does produce a type rich in beta-caryophyllene from the needles, cones, wood, and bark. Interestingly, researchers found that a chemotype devoid of beta-caryophyllene but rich in the sesquiterpene longifolene (33.1%) and also palmitic acid (9.3%) and longicamphenylone (7.9%) binds to CB2R [87].

Many of the aromatic compounds (listed in descending binding affinity: palmitic acid, 1-octadecanol, myristic acid, dodecanoic acid, 7 (11)-selin-4-alpha-ol, caryophyllene oxide, humulene-1,6-dien-3-ol, longicyclene, longifolene, longicamphenylone, myrtenal, alpha-pinene, terpinolene, and delta-3-carene) within the oil also readily bound to glucocorticoid receptors. The endocannabinoid system plays a complex and critical role in the regulation of glucocorticoid-mediated hypothalamic-pituitary-adrenal (HPA) axis negative feedback, which is the major neuroendocrine axis regulating homeostasis in mammals. Under basal conditions, glucocorticoids are released rhythmically to maintain homeostasis with both a circadian and ultradian pattern. The robust rhythm in circulating glucocorticoid levels is vital to the normal function of glucocorticoid target organs and the HPA axis response to stress. The disruption of these rhythms is associated with disease in humans [88]. Glucocorticoid hormones are rapidly synthesized from the adrenal gland in response to stress. Consequently, a rapid loss of AEA in the amygdala occurs, possibly through rapid induction of FAAH-mediated hydrolysis [89]. The magnitude by which AEA is degraded negatively correlates with the extent of HPA axis activation. Greater AEA loss elicits correspondingly greater increases in corticosterone secretion [89]. As such, proper ECS tone under steady-state conditions acts to suppress the excitatory activity induced by glutamate signaling in the basolateral nucleus of the amygdala. This data is evidence that the ECS plays a crucial role in HPA response. Another correlation between glucocorticoids and the ECS is the ability of glucocorticoids to rapidly induce 2-AG synthesis in the hypothalamus [90]. At the functional level, interest in glucocorticoid and ECS interactions is growing.

**Figure 6:**
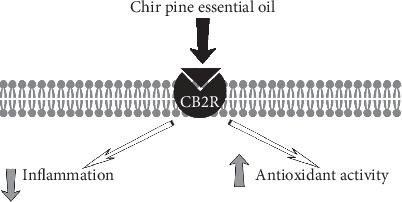
Chir pine essential oil displayed significant anti-inflammatory activity and improved superoxide dismutase and catalase activity.

Chir oil was administered (100 mg/kg body weight, p.o.) in mice, producing a significant anti-inflammatory activity and improved antioxidant activity (Figure 6), specifically elevation of superoxide dismutase and catalase activity. Superoxide dismutase and catalase are enzymes that protect cells from free radical assault by reactive oxygen species, specifically hydrogen peroxide and superoxide anion. Emerging evidence suggests that crosstalk between the ECS and reactive oxygen species signaling systems acts to modulate ECS functionality as well as redox homeostasis in different cell types [91]. The ECS is involved in regulation of reactive oxygen species generating and scavenging mechanisms and redox can modulate ECS function emphasizing an interplay between these two prominent signaling systems. In addition to binding to CB2R (Table 1), the oil activated *μ*, *δ*, and *κ* opioid receptors, suggesting that the oil may be beneficial for painful inflammatory conditions. It is possible that longifolene or other terpenoids present in the oil possess binding affinity for CB2R, or a biotransformation is occurring after ingestion to a compound with CB2R binding affinity. Biotransformation of longifolene to alcohols in mammals has been reported [92].

### 3.6. Wormwood (*Artemisia absinthium*) Essential Oil

Wormwood essential oil is not commonly used in aromatherapy and not widely available commercially. Preclinical research has identified that wormwood essential oil has pharmacological activities such as antioxidant, antimicrobial, and antiparasitic [93–95]. Absinthe is a potent alcoholic drink distilled from green anise, fennel, wormwood oil, and other herbs that has potential for both harm and abuse [96, 97]. The drink is believed to produce hallucinations with intense euphoria. Thujone is the active compound in wormwood essential oil, but also found in sage and tansy essential oils [98]. Speculating that thujone may interact with CB1R and result in absinthe's observed intoxicating effects, researchers investigated the effects of thujone on the ECS.

**Figure 7:**
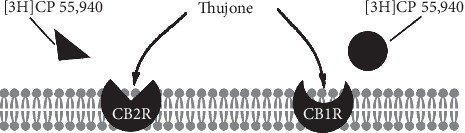
Despite weak binding affinity for CBR, higher concentrations of thujone displaced synthetic cannabinoid [3H]CP 55,940 from CB1R and CB2R, which may weakly affect ECS activity.

Using radioligand receptor binding assays and membrane preparation from rat brains containing CB1R and human tonsils with CB2R, the researchers demonstrated that thujone displaced the synthetic cannabinoid [3H]CP 55,940—a full agonist of CB1R and CB2R—only at higher concentrations than the synthetic (Figure 7) [99]. It had a very low binding affinity for CBR and failed to stimulate G-proteins even at 0.1 mM. In addition, rats administered thujone exhibited dissimilar behavior compared with rats administered levonantradol (a potent cannabinoid agonist). The data suggest that wormwood essential oil, rich in thujone, only weakly affects ECS function in higher concentrations (Table 1).

## 4. Discussion

Essential oils are complex mixtures of volatile organic compounds, which generally contain dozens to hundreds of low molecular weight compounds. In general, monoterpenes and sesquiterpenes are the predominant compounds of essential oils. Terpenes are a potential source of lipid modulators of the endogenous lipid systems, including the ECS, with the ability to stimulate cannabinergic signaling [28, 100, 101]. Some compounds can dominate the composition of the essential oil (such as methyl salicylate, which makes up more than 95% of genuine wintergreen oil), while others may be present in only fractions (such as incensole acetate in frankincense). Sometimes the main compound is responsible for the biological effects of the oil, but this is not always the case, and frequently greater activity is observed with the whole oil rather than isolated major compounds [2]. Among plant products, essential oils deserve particular attention for clinical use because of their use in numerous healing systems throughout the history.

Previous research has shown that essential oils with no known phytocannabinoids or CBR ligands in their composition may have ECS modulating properties, so as to attribute bioactivity entirely to a major compound ignores whole-plant chemistry and synergy. Plant biochemistry is complex and unique with minor compounds modulating or altering the effects of the main actives. One such example is myrcene—commonly found in hops, hemp, and lemongrass essential oils, which lacks antibacterial activity in isolation but enhances the antibacterial activity of other constituents like geranial and neral against Gram-positive and Gram-negative organisms [102]. Linalool is well-known for its effects on mood state, but even essential oils rich in this constituent, like rosewood, rely upon the synergy of minor constituents to produce antidepressant activity [103]. Terpinen-4-ol, the major constituent of genuine tea tree essential oil, possesses notable antimicrobial activity, [104–107] yet its anti-infectious (HSV-1) activity is a result of complex interactions among multiple constituents [108]. Similarly, the anticancer activity of mastic essential oil is attributed to the phytocomplexes of the whole essential oil rather than single constituents [109]. While instances of a single compound outperforming the whole essential oil exist, [110, 111] the buffering effects that may reduce toxicity or irritant capabilities of major active constituents cannot be ignored. Estragole—present in basil (*Ocimum basilicum*), fennel, and other essential oils—is considered carcinogenic but it may be inactivated by other molecules in the whole plant matrix [112]. Complex plant matrix effects require further study to confirm inactivation of toxicity or harm, from minor or major constituents, but ECS modulation by essential oils with no currently known cannabinoid suggests further research with whole essential oils is warranted.

The aforementioned studies suggest that essential oils modulate the ECS function in diverse ways both directly and indirectly. Direct binding to CB2R provides the means to modulate immune function and the inflammatory cascade. Direct agonists of CB2R allow for regulation of immune cells expressing CB2R (B cells, basophils, dendritic cells, eosinophils, mast cells, macrophages, NK cells, neutrophils, T cells, etc.) [9] and are prime targets for the treatment of various inflammatory conditions. CB2R play a critical role in alleviating perturbations involved in the pathogenesis of inflammatory conditions, ischemia/reperfusion injury, cancer, and neurodegenerative diseases [113, 114]. CB2R may work independently or cooperatively with CB1R in different cellular populations to regulate diverse biological and physiological functions in mental and physiological disorders. CB2R-selective agonists that do not generate undesirable psychoactive effects, like BCP, are increasingly appreciated from a therapeutic perspective. Myriad essential oils contain the direct selective agonist BCP, but copaiba is the leading essential oil for therapeutic use due to its significant levels.

Essential oils that modulate ECS activity represent novel therapeutics that could exert neuroprotective effects and slow disease progression in neurodegenerative disorders and conditions involving neuroinflammation, such as Alzheimer's disease, multiple sclerosis, and amyotrophic lateral sclerosis (ALS). Modified expression of CB1R and CB2R has been observed in a human brain with Alzheimer's disease [115–117]. Moreover, enhanced MAGL enzymatic activity and increased FAAH expression and activity have been observed postmortem in human Alzheimer's disease brain [118, 119]. These findings are suggestive that pathological changes in the ECS induced by inflammation may play a role in Alzheimer's disease. Activation of CB2R and neuroprotection by beta-caryophyllene combined with the reduction of FAAH and MAGL activity by lavender essential oil support a hypothesis that using these oils therapeutically may modulate the brain's innate response and reduce Alzheimer's disease progression.

Multiple sclerosis is an inflammatory demyelinating condition of the central nervous system, caused by autoimmune attack on myelin. Investigation of CBR in brain tissue samples from multiple sclerosis patients showed that CB1R- and CB2R-expressing cells are collected in active multiple sclerosis plaques and FAAH enzyme activity is induced [120]. AEA levels were strongly correlated with lesions visible on imaging. Other research suggests that endogenous endocannabinoid levels are disrupted in people with both active and silent multiple sclerosis lesions [121–124]. The totality of the research suggests that dysregulation of the ECS plays a role in the pathogenesis and progression of multiple sclerosis. The use of cannabimimetic essential oils to activate CB2R, particularly beta-caryophyllene, may help regulate myelination and promote immune balance, therefore slowing multiple sclerosis progression [27]. Direct binding to CB2R by copaiba and chir pine essential oils may help normalize endocannabinoid levels and reduce inflammatory and excitotoxic assault to myelin.

ALS causes degeneration of motor neurons in the cortex, brainstem, and spinal cord, resulting in progressive loss of voluntary muscle control. The pathology of ALS is associated with neuroinflammation, mostly triggered by excitotoxicity and oxidative assault [125–127]. Significantly increased CB2R-positive microglia and macrophages are noted in areas of motor neuron damage in ALS patients [128]. Dysregulation of the ECS seems to play a role in the pathogenesis of ALS, and reversing this dysregulation via CB2R could alter ALS progression. Here again, copaiba and chir pine, which act as direct CB2R agonists, are leading natural phytocompounds to investigate in ALS models.

Compounds that activate CB2R, expressed predominantly in immune cells, have neuroprotective and immunomodulatory activity that could improve the symptoms of several neuroinflammatory and neurodegenerative conditions. However, this requires further human studies to assess the effects of cannabimimetic essential oils and their effects on target receptors.

Indirect activity within the ECS—inhibition of FAAH and MAGL and activation of ECS in the CNS—represents another mechanism by which essential oils exert impactful positive modifications of the ECS and regulate human health. More research is necessary to determine which targets, mechanisms, or pathways within the ECS essential oils may regulate, in addition to direct binding to CB2R.

Multiple essential oils could be combined as a blend to modulate ECS activity, both directly and indirectly, to promote homeostasis and mitigate conditions whose pathogenesis involves dysregulation of the ECS due to the important regulatory functions of the ECS [129–131]. For example, copaiba is used for a direct CB2R agonist with lavender to inhibit FAAH. Furthermore, essential oils affect the biochemistry of our cells and have various biological properties (anti-inflammatory, antinociceptive, neuroprotective, anticancer, etc.) outside the ECS, which may create a synergistic or more complete resolution of root health problems. Studies have and continue to elucidate their mechanisms of action and pharmacological targets. The wide variety of compounds and distinctive mixtures of volatile compounds in essential oils means a great diversity in the mechanism of action, molecular targets, and epigenetic influence. Efforts need to be directed towards essential oil research in relation to the ECS and other pathways and mechanisms by which they improve physiology and biochemistry.

Based on effects from both inhalation and oral administration of essential oils, it is possible that essential oils activate specific areas of the brain or influence ECS regulation via olfactory receptors [132]. This is fascinating, considering olfactory receptors are expressed outside the nasal cavity and in vital organs like the heart, liver, lungs, kidneys, and gastrointestinal tract [133, 134].

Special consideration should be given to essential oils containing high concentrations of BCP such as copaiba, hemp, and melissa. Previous research has shown their substantial therapeutic value, making it clear that BCP-containing essential oils should be among the first phytomedicines investigated in relation to the ECS and human health and disease. In particular, BCP has exhibited influence on multiple pathways involved in cancer pathogenesis and chemoprotective properties. Given the growing threat cancer poses, research with BCP and cancer should be accelerated.

## 5. Limitations

This review has limitations. PubMed was the only database searched for relevant publications to be included in this review. Searches with other databases may have yielded additional results. The findings of this review are limited in that only experimental data are currently available regarding how EOs modulate the ECS activity. Human clinical trials are greatly needed to confirm experimental findings and advance the use of EOs for a variety of conditions involving dysregulation of the ECS.

## 6. Conclusion

Emerging evidence clearly suggests that essential oils may play a role in the regulation of the ECS, its enzymatic activity, and the production of endocannabinoids and therefore represent promising phytomedicines for the treatment of diseases and conditions involving dysregulation of the ECS. Further scientific study of additional essential oils' influence on the ECS, with an emphasis on those containing BCP, may reveal specific mechanisms of action by which essential oils modulate ECS regulatory activity. Once mechanisms are determined, human clinical trials must be conducted to determine appropriate usage and dosing in relation to the various human conditions and diseases that essential oils may benefit.

## Figures and Tables

**Table 1:** Essential oils and compounds with ECS activity.

Essential oil	ECS activity
Copaiba, guava leaf, black pepper, hemp, melissa, clove, ylang ylang^*∗*^	CB2R agonist

Lavender	Inhibits FAAH and MAGL enzymes

Cedarwood	Activates central CB1R and CB2R

Mastic	Increases cannabinoid congeners palmitoylethanolamide (PEA) and oleoylethanolamide (OEA)
Chir pine	CB2R agonist

Wormwood	Weakly affects ECS at higher concentrations

^*∗*^Essential oils that contain moderate to high levels of the active compound beta-caryophyllene.

## Abbreviations

AEA: N-Arachidonoylethanolamide
AKT: Protein kinase B
ALS: Amyotrophic lateral sclerosis
BCP: Beta-caryophyllene
CB1R: Cannabinoid receptor type 1
CB2R: Cannabinoid receptor type 2
CBD: Cannabidiol
CBR: Cannabinoid receptor
CNS: Central nervous system
ECS: Endocannabinoid system
COX-2: Cyclooxygenase-2 (COX-2)
DHA: Docosahexaenoic acid
EPA: Eicosapentaenoic acid
FAAH: Fatty acid amide hydrolase
HPA: Hypothalamic-pituitary-adrenal
iNOS: Inducible nitric oxide synthase
MAGL: Monoacylglycerol lipase
MAPK: Mitogen-activated protein kinase
mTOR: Mammalian target of rapamycin
OEA: Oleoylethanolamide
PEA: Palmitoylethanolamide
PI3K: Phosphoinositide 3-kinases
S6K1: Ribosomal protein S6 kinase beta-1
STAT3: Signal transducer and activator of transcription 3
THC: Δ9-Tetrahydrocannabinol.
